# Turpentine in Purpura Hemorrhagica

**Published:** 1846-04

**Authors:** 


5. *Turpentine in Purpura Hemorrhagica.*—The *Edinburgh Monthly Journal of Medical Science*, for December 1845, gives the history of two very interesting cases of purpura treated by large doses of turpentine. Dr. Neligan states that "it acts as a powerful cathartic, also possesses the property of checking hemorrhage, depending on an atonic state of the smaller blood-vessels, owing, probably, to its powers as a diffusible stimulant. In consequence of those views, I employed this remedy in four cases that afterwards came under my care while in charge of the district, and they all recovered. I prescribed the oil both in the form of draught and of enema; the usual dose for adults being from one ounce to an ounce and a half, and for children from two drachms to half an ounce, generally in combination with castor oil, to render its cathartic action more certain.

"Since that time I have employed oil of turpentine in every case of purpura which has been under my care, and its use has been invariably attended with beneficial results."—*N. Y. Jour. of Med. & Col. Sciences.*

